# Role of Sonazoid enhanced ultrasound assistant laparoscopic radiofrequency ablation in treating liver malignancy—A single‐center retrospective cohort study

**DOI:** 10.1002/cam4.5613

**Published:** 2023-03-06

**Authors:** Jingyong Xu, Yuan Liu, Mingxiao Wu, Jinghai Song, Chen Li

**Affiliations:** ^1^ Department of General Surgery, Department of Hepatobiliopancreatic Surgery, Beijing Hospital, National Center of Gerontology Institute of Geriatric Medicine, Chinese Academy of Medical Sciences Beijing China; ^2^ Department of Ultrasound, Beijing Hospital, National Center of Gerontology, Institute of Geriatric Medicine Chinese Academy of Medical Sciences Beijing China

**Keywords:** contrast‐enhanced ultrasound, laparoscopy, liver malignance, radiofrequency ablation, Sonazoid

## Abstract

**Aim:**

To evaluate the role of Sonazoid enhanced ultrasound assistant laparoscopic radiofrequency ablation in treating liver malignancy.

**Methods:**

Consecutive patients are recruited. Rates of complication and postoperative length of stay are compared between the study and control groups. Progression‐free survival (PFS) of colorectal liver metastasis (CRLM) after ablation are compared. Complete ablation rates are compared and optimal tumor size is calculated by ROC curve analysis. Risk factors of incomplete ablation are determined by logistic regression analysis.

**Results:**

Totally 73 patients with 153 lesions were included. No significant differences in the rate of complication were found between the study and control groups. PFS of CRLM in laparoscopic, intraoperative CEUS, and laparoscopic CEUS groups are all longer than their control groups. Complete ablation rates of laparoscopic, intraoperative CEUS, and laparoscopic CEUS groups are all higher than in their control groups with statistical significance. A tumor size of 2.15 cm is determined to be the optimal cut‐off with the area under the ROC curve of 0.854, 95% CI (0.764, 0.944), *p* = 0.001. In logistic regression analysis, tumor size [OR 20.425, 95% CI (3.136, 133.045), *p* = 0.002] and location of segments VII and VIII [OR 9.433, 95% CI (1.364, 65.223), *p* = 0.023] are calculated to be the risk factors of incomplete ablation, meanwhile, intraoperative CEUS shows to be a protective factor in univariate analysis [OR 0.110, 95% CI (0.013, 0.915), *p* = 0.041].

**Conclusion:**

Sonazoid‐enhanced ultrasound assistant laparoscopic radiofrequency ablation is safe and effective to treat liver malignancy. We should pay attention to the ablation planning of larger tumors and tumors in special locations.

## Graphical abstract

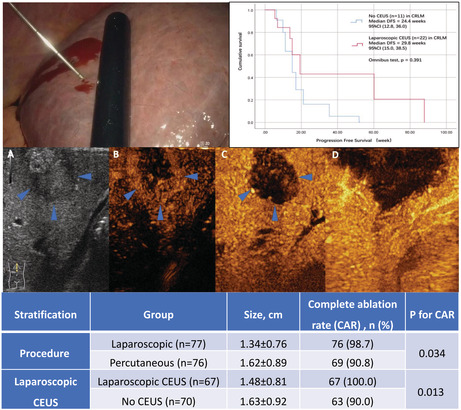

## 1 INTRODUCTION

Hepatic malignancies, including primary liver cancer (hepatocellular carcinoma, cholangiocarcinoma, combined hepatocellular‐cholangiocarcinoma) and hepatic metastasis from primaries including colorectal, pancreas, and breast, were characterized by a high degree of malignancy, fast growth, easy to metastasis and invasion, and accompanied serious complications in the late stage. Radiofrequency ablation (RFA) is one of the most widely used minimally invasive techniques, which is proven to be one of the therapeutic strategies besides surgical resection.^1^, ^2^, ^3^

Intraoperative contrast‐enhanced ultrasound (IO‐CEUS) has been confirmed to be more sensitive than intraoperative gray ultrasound (IOUS) in detecting malignant tumors and subsequently changing surgical strategy.^4^, ^5^ Contrast agent plays an important role. Sonazoid is a special contrast agent which is different from traditional ones. Due to the parenchyma‐specific accumulation of Sonazoid in the Kupffer cells, a late Kupffer‐phase image can be achieved and in this phase, almost all tumors show a “black hole sign” and are easy to detect. We also had more than 1 h to scan the whole liver under the contrast background.^6^ Our previous study showed that the sensitivity of IO‐CEUS using the Kupffer phase in identifying hepatic malignant tumors was the highest among the radiologic approaches and laparoscopic CEUS was proved to be the best choice as it possessed a trend of changing the treatment strategy toward a higher curative rate.^5^ While most published studies focused on the high effect of detecting and identifying malignant tumors by intraoperative CEUS during surgery, the results of survival status are still uncertain.^7^ What is more is that, fewer studies of CEUS using Sonazoid and Kupffer phase were published.

Therefore, we designed this study to evaluate whether Sonazoid and its Kupffer phase are helpful to assist laparoscopic RFA in treating liver malignancies, to get better short‐ and long‐term outcomes.

## 2 METHODS

### 2.1 Patients and baseline characteristics

Totally, we treated 115 patients consecutively by RFA from June 2019 to September 2021 with 218 lesions. We selected patients according to the following inclusion criteria: (1) malignant diseases including hepatocellular carcinoma (HCC) and colorectal liver metastasis (CRLM) but not recurrent tumors, (2) all lesions were detectable and countable by preoperative ultrasound, (3) maximal diameter of single lesion ≤5 cm, (4) percutaneous and laparoscopic ablation but not an open procedure, and (5) complete postoperative follow‐up record for at least 6 months. Patients are then grouped in different ways: (1) laparoscopic and percutaneous groups, (2) contrast‐enhanced ultrasound (CEUS) and no CEUS groups, (3) laparoscopic CEUS and no CEUS groups, and (4) laparoscopic CEUS and no CEUS groups in treating lesions in segments VII and VIII, which were considered as difficult‐to‐treat tumors.^8^ According to the purpose of this study, we chose the baseline characteristics as follows: age, gender, preoperative liver function (ALT and total bilirubin), number of local lesions in the liver, postoperative adjuvant therapy, pathology, and amount of simultaneous colorectal resection and liver lesion resection.

### 2.2 Study design

**FIGURE 1:**
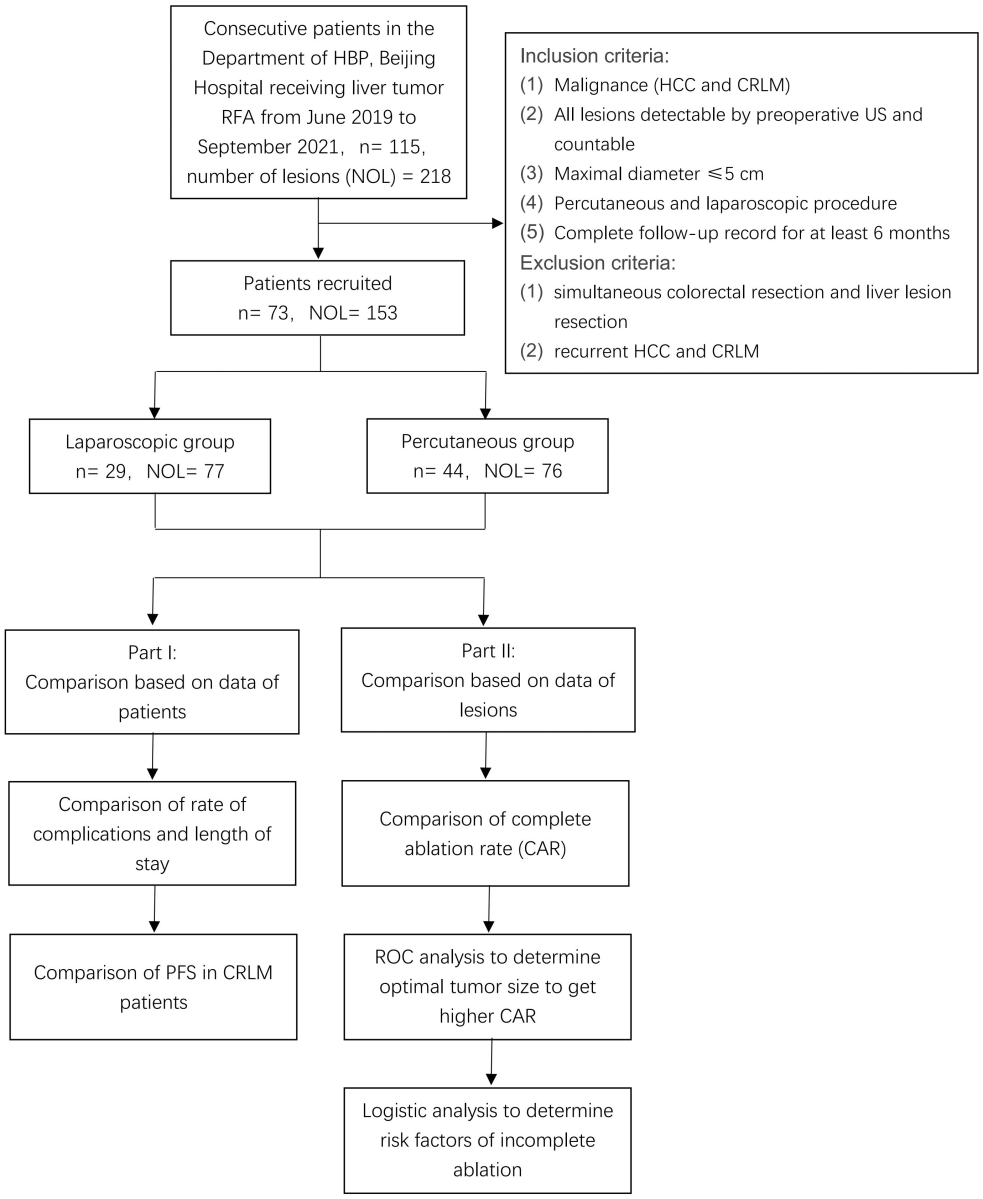
Flowchart of the study

The study contained two parts. The first part was based on the analysis of data from patients, including the comparisons of basal data, operation‐associated complications, and length of stay. Then the comparison of PFS was conducted in CRLM patients. The second part was based on the analysis of data from tumors, including comparisons of complete ablation rate (CAR), calculation of optimal tumor size to get higher CAR, and determination of the risk factors of incomplete ablation. Figure 1 displays the study design.

The ethics committee of Beijing Hospital approved the usage and publication of these data and written informed consents were signed by patients before the operation (Ethic approval letter No. 2021BJYYEC‐190‐02).

### 2.3 Operative procedures

In this study, only laparoscopic and percutaneous procedures were included. Since this was a real‐world study, all treatment strategies were determined by the multidiscipline team (MDT) in Beijing Hospital including physicians from departments of gastrointestinal surgery, hepatobiliary surgery, oncology, radiology, and ultrasound. The surgeons determined the surgical route by their experience and both surgeons and ultrasound physicians had more than 10 years of experience and received unified and standardized training in our department.

**FIGURE 2:**
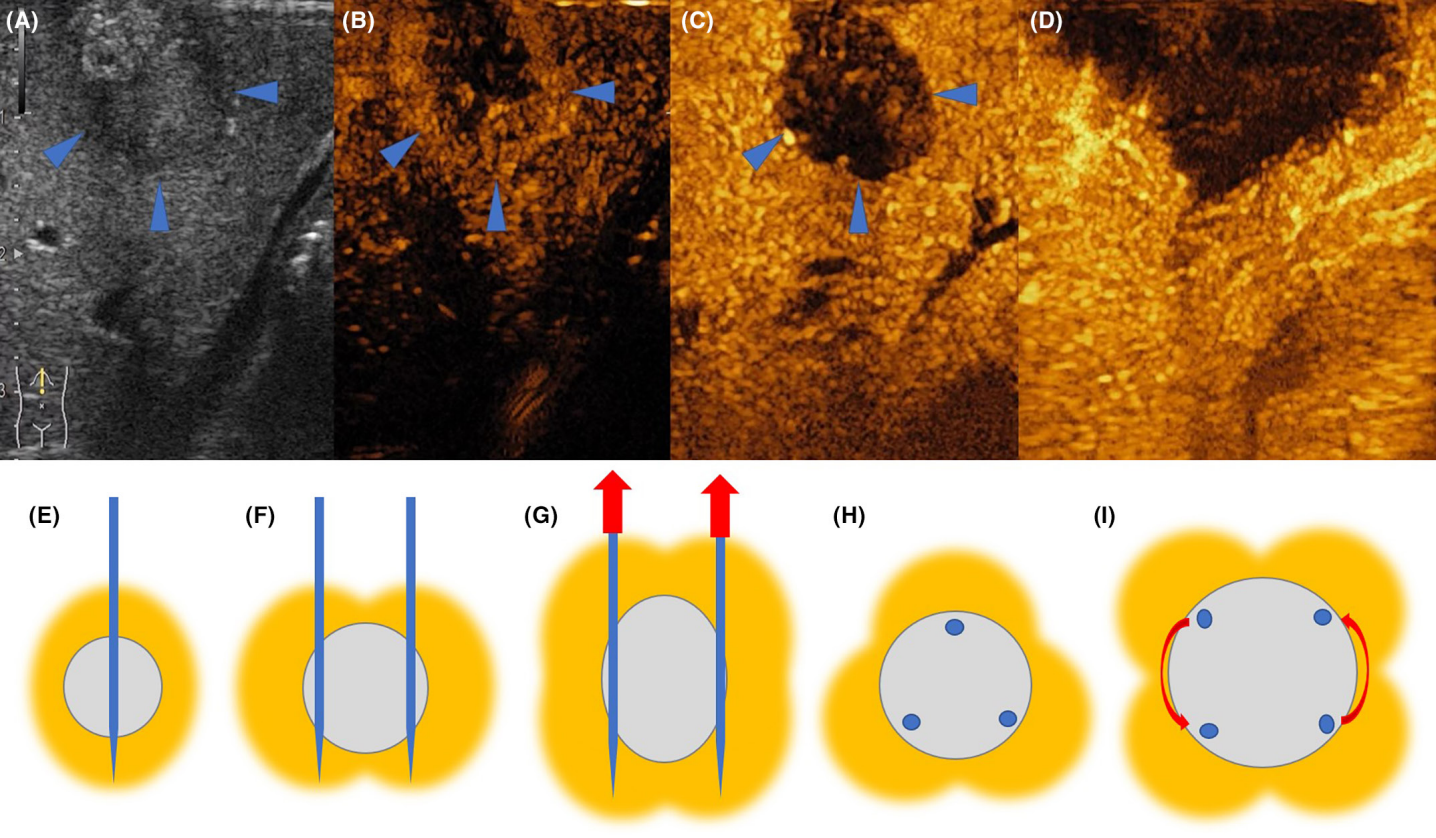
CEUS images and diagram of the arrangement of the needles. (A) Gray scale. (B) Arterial phase. (C) Kupffer phase. (D) reperfusion after ablation. Blue arrows outline the tumor. (E) one‐needle plan. For tumors <1.5 cm. (F) Two‐needle plan. For round tumors with diameter of 1.5–2.5 cm. (G) Two‐needle plan with pulling‐back skill. For oval tumors with the longest diameter beyond 3 cm or large tumors (3–5 cm). (H) Three‐needle plan. For large tumors. Often combine with pulling‐back skill. (I) two‐needle plan with rotation. For large tumors. Often combine with pulling‐back skill

In this study, we used perfluorobutane microbubbles (Sonazoid, GE Healthcare, Oslo, Norway) as the contrast agent in the CEUS. Sonazoid can be specially taken in by Kupffer cells in the liver and provides a post‐vascular phase 10 min after the vascular phase called Kupffer phase which can last more than 1 h.^6^ In the vascular phase, the ultrasound images of HCC and CRLM are different, which are the basis of diagnosis and differentiated diagnosis. However, in the Kupffer phase, there is little difference between HCC and CRLM images, both of which are hypoechoic compared with the hyperechoic background of the normal liver (black hole signs) (Figure 2C). In this study, the ablations in both laparoscopic and percutaneous operations with IO‐CEUS of HCC and CRLM were done under the guidance of Kupffer phase. We injected the contrast intravenously at least 10 min before sterilizing and did the whole liver examination and tumor ablation in Kupffer phase. A core‐needle biopsy was routinely done before ablation.

All the patients recruited in this study underwent RFA with 3‐cm Cool‐tip applicators. One to three needles were used depending on the size and shape of the tumor to ensure a sufficient ablating edge of at least 0.5 cm around the tumor. Reperfusion was done 10–15 min after the ablation (Figure 2D). The strategy of the arrangement of RFA needles is shown in Figure 2E–I and the strategies to treat HCC and CRLM were the same.

### 2.4 Follow‐up and evaluation strategies

The follow‐up was done by the same physician team according to the recent guidelines.^9^ We defined residual lesion as evidence of active tumor in the ablated focus in any two radiologic exams including CEUS, enhanced CT, enhanced MRI, and PET‐CT 1 month after the ablation, thereafter, the complete ablation rate is recorded as the major outcome of this study. Since the ablation and assessment strategies of HCC and CRLM were the same, which contained at least 0.5 cm margin, way of arrangement of ablation needle (Figure 2E–I), and way of defining residue lesion. So when the CARs were compared between different groups, we put the data of ablation ranges from both HCC and CRLM lesions together to analyze, which might not be influenced by the pathology.

Major complications included: (1) operation‐related ones, such as hemorrhage, liver abscess, biloma, skin burn, and wound infection, and (2) systemic ones, such as anesthetic complications and plural diffusion. We also compared the postoperative length of stay (LOS).

We only analyzed progression‐free survival (PFS) in CRLM patients as the amount of laparoscopic operation of HCC was too small (*n* = 7). PFS is defined as the duration between complete ablation and progression and is counted by week in this study. Progression contains three clinical conditions: (1) in situ recurrence which is defined as active tumor evidence in the ablated lesion 3 months after the operation, (2) newly onset lesion, and (3) metastasis. We analyzed the PFS of CRLM after ablation through three stratifications: (1) laparoscopic and percutaneous groups, (2) intraoperative CEUS and no CEUS groups, and (3) laparoscopic CEUS and no CEUS groups.

### 2.5 Statistical analysis

Two staff collected and checked the data to ensure accuracy. IBM SPSS Statistics (Ver. 26.0, IBM Corp., Armonk, NY, USA) was used for statistical analysis. Categorical data were analyzed by the chi‐square test or the Fisher's exact test. Continuous data were tested using Student's unpaired *t*‐test. The diameter of lesions was expressed by the median and interquartile range (IQR), and compared by the Mann–Whitney *U* test. The survival was mentioned by the Kaplan–Meier method, and survival rates were compared by log‐rank test. If the survival curves cross each other, we added Cox regression analysis to balance the varieties. The risk factors were determined according to clinical risk score (CRS), containing CEA, and lesion number in the liver. Age, procedure, and postoperative adjuvant therapy were also included. ROC curve was shown to define the cut‐off of the diameter of the lesion according to the complete ablation rate. Multivariant logistic regression analysis was used to evaluate the relationship between risk factors and complete ablation rate, which is shown as an odds ratio (OR) with 95% confidence intervals. Factors with a *p* value < 0.1 in the univariant regression analysis were chosen to enter the multivariable analysis. We defined the risk factors by referring to the literature and our database, including size, location, procedure, intraoperative CEUS, and pathology.^10^, ^11^ *p* Values of <0.05 were determined statistically significant.

## 3 RESULTS

### 3.1 Part I: Comparison based on patient data

#### 3.1.1 Basal characteristics

Totally, 73 patients were recruited in the study. The comparisons of basal data are displayed in Table 1. The number of lesions in the laparoscopic group is more than in the percutaneous group with statistical significance, meanwhile, the proportion of pathology is also significantly different. To explain these differences, we did a further subgroup analysis based on the pathology. The number of tumors in the CRLM group is significantly more than in the HCC group [2.0 (2.5) vs. 1.0 (0.0), *p* = 0.002]. The surgeons prefer the laparoscopic route in treating CRLM [53.5% (22/41) vs. 24.1% (7/29), *p* = 0.006]. From the results, we could infer that the differences in basal data were due to the pathologic characteristics and the treatment methods. Since this study is an evaluation of the effect of the operation, these differences do not affect the interpretation of the results.

**TABLE 1:** Comparison of basal data and outcomes

	Laparoscopic (*n* = 29)	Percutaneous (*n* = 44)	*p*	Intraoperative CEUS (*n* = 33)	No CEUS (*n* = 40)	*p*
Basal data						
Age, year	61.2 ± 10.8	64.3 ± 11.5	0.248	59.4 ± 9.4	66.1 ± 11.9	0.011
Sex (Male), *n* (%)	23 (79.3)	33 (75.0)	0.670	24 (72.7)	32 (80.0)	0.464
TBIL, μmol/L	17.1 ± 17.4	15.2 ± 11.3	0.574	16.5 ± 16.3	15.5 ± 11.9	0.768
ALT, U/L	35.7 ± 44.4	51.3 ± 72.8	0.264	43.9 ± 59.4	35.8 ± 38.1	0.483
Number of lesions, median (IQR)	2.0 (3.0)	1.0 (0.0)	0.001	1.0 (1.0)	1.0 (1.5)	0.289
Adjuvant therapy, *n* (%)	21 (72.4)	29 (65.9)	0.558	25 (75.8)	25 (62.5)	0.225
Pathology (HCC), *n* (%)	7 (24.1)	25 (56.8)	0.006	9 (27.3)	23 (57.5)	0.010
Spontaneous laparoscopic liver or colorectal resection			<0.001			0.038
Percutaneous ablation, *n* (%)	0 (0.0)	35 (79.5)		11 (33.3)	24 (60.0)	
Laparoscopic ablation, *n* (%)	14 (48.3)	0 (0.0)		9 (27.3)	5 (12.5)	
Spontaneous liver resection, *n* (%)	6 (20.7)	0 (0.0)		6 (18.2)	0 (0.0)	
Spontaneous colorectal resection, *n* (%)	3 (10.3)	8 (18.2)		3 (9.1)	8 (20.0)	
Spontaneous liver and colorectal resection, *n* (%)	6 (20.7)	1 (2.3)		4 (12.1)	3 (7.5)	
Outcomes						
Major complications, *n* (%)	0 (0)	3 (6.8)	0.272	1 (3.0)	2 (5.0)	0.573
LOS, day	6.7 ± 2.9	4.2 ± 3.4	0.001	5.6 ± 3.2	4.9 ± 3.6	0.351

Abbreviations: ALT, alanine transaminase; CEUS, contrast enhanced ultrasound; HCC, hepatocellular carcinoma; IQR, interquartile range; LOS, length of stay; TBIL, total bilirubin.

#### 3.1.2 Comparison of outcomes

Table 1 also shows the results of comparisons of two major outcomes between groups. No significant differences in the rate of complications are found between the two groups. Only in the comparison of the length of postoperative stay between laparoscopic and percutaneous groups, the result shows significantly different (laparoscopic group 6.7 ± 2.9 vs. percutaneous group 4.2 ± 3.4).

#### 3.1.3 Comparisons of PFS in CRLM

**FIGURE 3:**
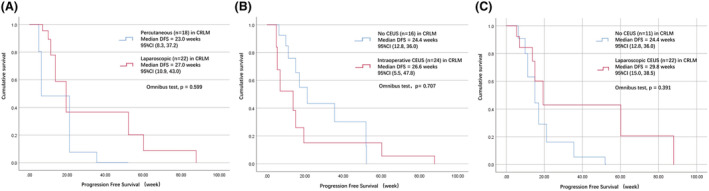
Comparisons of PFS. (A) Between laparoscopic and percutaneous groups. (B) Between intraoperative CEUS and no CEUS groups. (C) Between laparoscopic CEUS and no CEUS groups

Survival analysis should base on pathology. Due to the small number of cases in the laparoscopic HCC group, we only analyzed CRLM by three stratifications. Figure 3 shows that the PFS of the laparoscopic group, intraoperative CEUS group. and laparoscopic CEUS group are all longer than in the control groups, especially in the laparoscopic CEUS group, we can see 5.4 weeks longer. Although there is no statistical difference, from a clinical point of view, we still recommend laparoscopic CEUS‐guided radiofrequency for the treatment of CRLM.

### 3.2 Part II: Comparison based on lesion data

#### 3.2.1 Analyses of complete ablation rate

Totally 153 lesions were analyzed in 73 patients. The average size is 1.4 ± 0.8 cm (range: 0.4–4.9). The major outcome is CAR. We studied it in a variety of groups. Table 2 shows the results. All the Ps of the size and location distribution comparisons in the four stratifications are more than 0.05, which means the basal data are comparable. Meanwhile, the CARs in laparoscopic, CEUS, laparoscopic CEUS, and laparoscopic CEUS in Seg. VII and VIII groups are all statistically higher than CARs in the control groups.

**TABLE 2:** Comparisons of complete ablation rate between different groups

Stratification	Group	Size, cm	Location (Seg.VII–VIII), *n* (%)	CAR, *n* (%)	*p* For CAR
Procedure	Laparoscopic (*n* = 77)	1.34 ± 0.76*	33 (42.9)**	76 (98.7)	0.034
	Percutaneous (*n* = 76)	1.62 ± 0.89	28 (36.8)	69 (90.8)	
CEUS	Intraoperative CEUS (*n* = 83)	1.34 ± 0.84***	36 (43.4)^****^	82 (98.8)	0.024
	No CEUS (*n* = 70)	1.63 ± 0.92	25 (35.7)	63 (90.0)	
Laparoscopic CEUS	Laparoscopic CEUS (*n* = 67)	1.48 ± 0.81^*****^	30 (44.8)^******^	67 (100.0)	0.013
	No CEUS (*n* = 70)	1.63 ± 0.92	25 (35.7)	63 (90.0)	
Lesions in Seg. VII‐VIII	Intraoperative CEUS (*n* = 36)	1.20 ± 0.73^*******^	一	36 (100.0)	0.003
	No CEUS (*n* = 25)	1.47 ± 0.97	一	19 (76.0)	

Abbreviations: CAR, complete ablation rate; CEUS, contrast enhanced ultrasound; Seg., segment.

* *p* = 0.151

** *p* = 0.447

*** *p* = 0.187

^****^ *p* = 0.335

^*****^ *p* = 0.116

^******^ *p* = 0.279

^*******^ *p* = 0.226.

#### 3.2.2 Calculation of cut‐off value of optimal size

**FIGURE 4:**
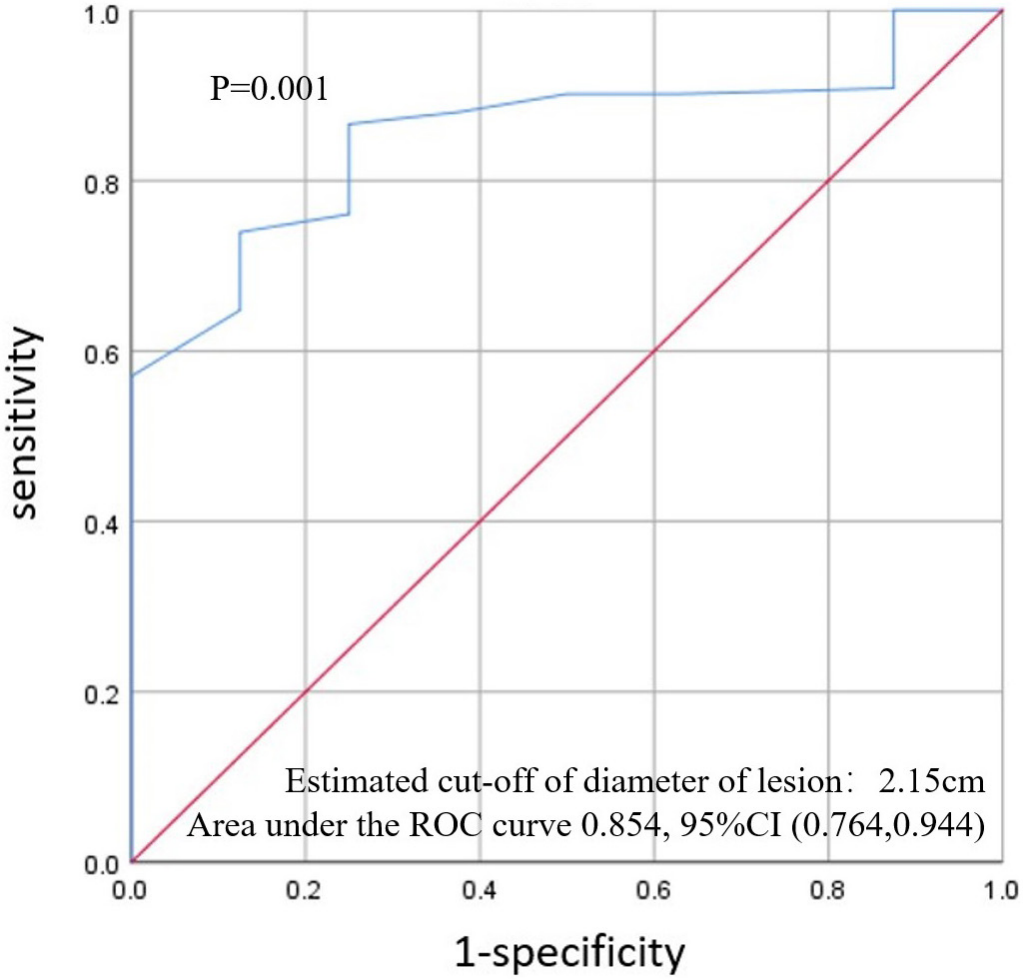
ROC curve for the estimation of the cut‐off values of tumor size

The total complete ablation rate is 94.8% (145/153). Taking this variable as a reference, we used the ROC curve to calculate the reference value of tumor size to obtain the optimal complete ablation rate. The results show that 2.15 cm is the best cut‐off value, that is, tumors less than 2.15 cm will obtain a higher complete ablation rate than larger tumors. The details are shown in Figure 4.

#### 3.2.3 Determination of risk factors of complete ablation rate

According to the cut‐off value of tumor size, the lesions were divided into greater than 2.15 group and less than 2.15 group. Logistic regression was used to set univariate analysis, and those with *p* < 0.1 entered multivariate analysis. The results are shown in Table 3. It can be seen that size and location are independent risk factors for incomplete ablation. There is no significant difference in procedure and intraoperative CEUS.

**TABLE 3:** Logistic regression analysis of risk factors of complete ablation rate

	Univariate OR (95% CI)	*p*	Multivariate OR (95% CI)	*p*
Diameter >2.15 cm	19.421 (3.650, 103.337)	0.001	20.425 (3.136, 133.045)	0.002
Intraoperative CEUS	0.110 (0.013, 0.915)	0.041	0.182 (0.010, 3.187)	0.243
Lesion location (Seg. VII–VIII)	4.909 (0.957, 25.183)	0.056	9.433 (1.364, 65.223)	0.023
Procedure (laparoscopic)	0.130 (0.016, 1.081)	0.059	0.587 (0.036, 9.575)	0.708
Pathology	2.718 (0.648, 11.399)	0.172		

## 4 DISCUSSION

There are multiple RFA methods for ablation modality, including percutaneous, open, and laparoscopic routes. Each route has its pros and cons. Percutaneous and laparoscopic RFAs are preferred as minimally invasive techniques, especially for small lesions, deep locations, multiple histories of abdominal operation, and complex co‐morbidity. Laparoscopic‐RFA (L‐RFA) is an alternative when percutaneous RFA (P‐RFA) is not feasible, for example, the tumor is near other organs or the tumor locates in higher or deeper segments including segments VII, VIII, and I. Many studies proved the efficiency and safety of ablation alone or combined with resection and adjuvant therapy for the management of hepatic malignancies.^12^ Ultrasound, especially intraoperative CEUS, is an effective tool guiding real‐time monitoring for ablation to achieve a precise approach and ablation of the tumor.

Sonazoid is a special contrast agent which can provide a long duration of contrast imaging called Kupffer phase or post‐vascular phase. In this phase, both typical images of HCC and CRLM are hypoechoic in a hyperechoic liver background. Sonazoid was reported to be safe and Sonazoid‐enhanced CEUS could be performed safely.^13^ Our study manifested that no significant difference in the rate of complications was found between laparoscopic and percutaneous groups as well as IO‐CEUS and no CEUS groups. LOS of the laparoscopic group was a little longer than that of the percutaneous group (6.7 ± 2.9 vs. 4.2 ± 3.4), which is due to the relatively complicated process of the laparoscopic operation.

Our study separately investigated the treatment of laparoscopic CEUS and L‐RFA for CRLM. Compared to percutaneous and no CEUS groups, we can see longer PFS in the laparoscopic group, intraoperative CEUS group, and laparoscopic CEUS group, especially in the laparoscopic CEUS group, though there is no statistical difference. Bartoș et al. demonstrated that local recurrence was lower in the laparoscopic group than in the percutaneous group in the study of ablation of liver metastases, which was consistent with our views.^14^ However, RFA is only a part of the comprehensive treatment of CRLM, which is not a local disease but a systemic disease. Although almost all the patients with liver metastases get to the status of no evidence of disease (NED) by RFA, local recurrence and metastasis are more affected by tumor biological characteristics and the response of systemic treatment. So it is difficult to get better even statistically better survival just by this procedure, especially in this retrospective study with bias that is hard to control. For HCC, Eun et al. indicated that the efficacy of laparoscopic‐RFA was superior to percutaneous‐RFA in terms of survival for HCC patients untreated for surgery.^15^ Therefore, the efficacy of RFA for laparoscopic HCC may be more meaningful, but it is a pity that there is no further analysis to form the cohort in our study due to the small number of HCC cases the in laparoscopic group.

Besides the discussion based on disease, we preferred to talk about the technique and assess the value of Sonazoid or Kupffer phase‐guided CEUS in tumor ablation. The complete ablation rate is the main outcome. From our statistical data of each group, the laparoscopic CEUS group improved the complete ablation rate. As ultrasound agents can detect vessels as small as 40 microns, they may detect residual neoplastic tissue after RFA.^16^ The Kupffer phase of intraoperative CEUS could be used to conduct real‐time monitoring in L‐RFA, and the efficiency of defect reperfusion imaging of intraoperative CEUS could be proved immediately after RFA.^17^ However, L‐RFA is a technically demanding operation. First, the exposure of tumors in difficult positions, such as segments VII, VIII, and I, could be challenging and need hepatic mobilization, and tumors in these difficult locations may often be excluded by some high‐quality studies^18^; second, it is hard to puncture the tumor following the trajectory in coordination with laparoscopic intraoperative ultrasound.^19^, ^20^ Therefore, the localization of laparoscopic intraoperative ultrasound required experienced doctors and possessed a learning curve. In this study, all recruited operations were completed by the same surgeon and his team to ensure consistent quality. Hence the complete rate of laparoscopic RFA guided by CEUS reached 100%.

A tumor size of 3 cm is determined to be the optimal cutoff in the major literatures and ≤3 cm was recommended for ablation in both clinical HCC and CRLM guidelines.^21^, ^22^ However, our study calculated a new cut‐off value, 2.15 cm, by ROC analysis. We are not challenging the widely accepted criteria, but we set a range, of 2.15–3 cm, to remind physicians to pay more attention when making the ablation plan. In terms of common sense, the smaller the tumor is, the greater the possibility of a satisfactory surgical margin could be. A range of 0.5–1 cm margin is recommended in major guidelines. It means that if we want to ablate a 3 cm tumor completely, we should make at least 4 cm damage of tissue wrapping the tumor. However, in clinical practice, it is difficult to puncture the tumor just in the middle, so this technological defect leads to the risk of residue lesions. In terms of instrument and technology, although the range is controllable due to the energy limitation of the radiofrequency needle, it also limits the complete ablation of larger tumors, especially in the vicinity of large blood vessels. In some studies and guidelines, the authors recommended using microwave ablation to make a bigger ablating range, which may reach 5 cm.^23^, ^24^ In our center, we have developed a strategy of needle arrangement according to the tumor size and shape to ensure the margin (Figure 2), containing a “pulling‐back” skill, multi‐point rotation skill, and multi‐needle arrangement. In terms of the pathology of liver malignancy, the endpoint and purpose of treatment are different in HCC and CRLM. In HCC, we aimed to get a curative effect, since most HCCs are local diseases. Some guidelines recommended 2 cm as a cut‐off in treating a single HCC by RFA.^25^ For 2–3 cm HCC, some authors recommended multi‐modular therapy like combined transarterial chemoembolization (TACE) and RFA to get a lower local tumor progression rate.^26^ For CRLM, the criterion is still 3 cm. Due to relatively late tumor staging, the effect of treatment depends on systematic therapy. Therefore, we should pay attention to the arrangement of the ablation needles for more than 2.15 cm malignant tumors, or we should add multi‐module therapy to ensure the treatment effect.

Although there is no significant difference in the regression analysis of procedure and IO‐CEUS, the complete ablation rates in the laparoscopic group and CEUS group are significantly higher than those in their control groups, especially in multiple CRLM and the tumors in a special location, so we also recommend the laparoscopic approach and IO‐CEUS.^11^, ^27^

The main limitations of this study include a single‐center, retrospective design and a relatively small sample size. A multi‐center study with a larger sample based on our study has been registered and ready to carry out (ChiCTR2200065890).

## 5 CONCLUSION

Kupffer phase‐based or Sonazoid enhanced ultrasound assistant laparoscopic RFA is a safe and effective procedure to treat liver malignancies. Laparoscopic CEUS improved the complete ablation rate. We should pay attention to the RFA planning of larger malignant tumors and tumors in segments VII and VIII and laparoscopic CEUS‐assisted RFA could be the first choice.

## AUTHOR CONTRIBUTIONS

**Jingyong Xu:** Conceptualization (lead); data curation (lead); formal analysis (lead); funding acquisition (lead); investigation (lead); methodology (lead); project administration (lead); resources (lead); software (equal); supervision (lead); validation (equal); visualization (equal); writing – original draft (lead); writing – review and editing (lead). **Yuan Liu:** Conceptualization (equal); data curation (equal); formal analysis (equal); investigation (equal); methodology (equal); writing – review and editing (equal). **Mingxiao Wu:** Conceptualization (equal); data curation (equal); formal analysis (equal); investigation (equal); methodology (equal); writing – review and editing (equal). **Jinghai Song:** Conceptualization (equal); methodology (equal); project administration (equal); writing – review and editing (equal). **Chen Li:** Conceptualization (equal); data curation (equal); formal analysis (equal); funding acquisition (equal); investigation (equal); methodology (equal); project administration (equal); writing – original draft (equal); writing – review and editing (equal).

## FUNDING INFORMATION

This study was supported by National High Level Hospital Clinical Research Funding (No. BJ‐2022‐128) from Jingyong Xu and Beijing Hospital Project (No.BJ‐2021‐187) from Chen Li.

## CONFLICT OF INTEREST

No benefits in any form have been received or will be received from a commercial party related directly or indirectly to the subject of this article. The authors declare no conflict of interest.

## ETHICAL APPROVAL

This study was approved by the Ethics Committee of the Beijing Hospital (2021BJYYEC‐190‐02).

## Data Availability

The data that support the findings of this study are available from the corresponding author upon reasonable request

## References

[1] Livraghi T , Meloni F , Di Stasi M , et al. Sustained complete response and complications rates after radiofrequency ablation of very early hepatocellular carcinoma in cirrhosis: is resection still the treatment of choice? Hepatology. 2008;47(1):82‐89. 18008357 10.1002/hep.21933

[2] Livraghi T , Solbiati L . Percutaneous treatment: radiofrequency ablation of hepatic metastases in colorectal cancer. Tumori. 2001;87(1 Suppl 1):S69. 11300032

[3] Dietrich CF , Lorentzen T , Appelbaum L , et al. EFSUMB guidelines on interventional ultrasound (INVUS), part III – abdominal treatment procedures (short version). Ultraschall Med. 2016;37(1):27‐45. 26871408 10.1055/s-0035-1553965

[4] Torzilli G , Del Fabbro D , Palmisano A , et al. Contrast‐enhanced intraoperative ultrasound during hepatectomies for colorectal cancer liver metastases. J Gastrointest Surg. 2005;9(8):1148‐1153. discussion 1153–1154. 16269386 10.1016/j.gassur.2005.08.016

[5] Li C , Liu Y , Xu J , Song J , Wu M , Chen J . Contrast‐enhanced intraoperative ultrasound with Kupffer phase may change treatment strategy of metastatic liver tumors – a single‐Centre prospective study. Ther Clin Risk Manag. 2021;17:789‐796. 34366666 10.2147/TCRM.S317469 PMC8337051

[6] Kudo M . Defect reperfusion imaging with Sonazoid®: a breakthrough in hepatocellular carcinoma. Liver Cancer. 2016;5(1):1‐7. 26989655 10.1159/000367760 PMC4789887

[7] Loss M , Schneider J , Uller W , et al. Intraoperative high resolution linear contrast enhanced ultrasound (IOUS) for detection of microvascularization of malignant liver lesions before surgery or radiofrequeny ablation. Clin Hemorheol Microcirc. 2012;50(1–2):65‐77. 22538536 10.3233/CH-2011-1444

[8] Schullian P , Putzer D , Laimer G , Levy E , Bale R . Feasibility, safety, and long‐term efficacy of stereotactic radiofrequency ablation for tumors adjacent to the diaphragm in the hepatic dome: a case‐control study. Eur Radiol. 2020;30(2):950‐960. 31489472 10.1007/s00330-019-06399-y PMC6957558

[9] Solbiati L , Ierace T , Tonolini M , Cova L . Guidance and monitoring of radiofrequency liver tumor ablation with contrast‐enhanced ultrasound. Eur J Radiol. 2004;51(Suppl):S19‐S23. 15311434 10.1016/j.ejrad.2004.03.035

[10] Schullian P , Johnston E , Laimer G , et al. Frequency and risk factors for major complications after stereotactic radiofrequency ablation of liver tumors in 1235 ablation sessions: a 15‐year experience. Eur Radiol. 2021;31(5):3042‐3052. 33125554 10.1007/s00330-020-07409-0 PMC8043912

[11] Takahashi H , Berber E . Role of thermal ablation in the management of colorectal liver metastasis. Hepatobiliary Surg Nutr. 2020;9(1):49‐58. 32140478 10.21037/hbsn.2019.06.08 PMC7026789

[12] van Amerongen MJ , van der Stok EP , Fütterer JJ , et al. Short term and long term results of patients with colorectal liver metastases undergoing surgery with or without radiofrequency ablation. Eur J Surg Oncol. 2016;42(4):523‐530. 26856957 10.1016/j.ejso.2016.01.013

[13] Itabashi T , Sasaki A , Otsuka K , Kimura T , Nitta H , Wakabayashi G . Potential value of sonazoid‐enhanced intraoperative laparoscopic ultrasound for liver assessment during laparoscopy‐assisted colectomy. Surg Today. 2014;44(4):696‐701. 23670037 10.1007/s00595-013-0607-4 PMC3950561

[14] Bartoș A , Bartos D , Spârchez Z , et al. Laparoscopic contrast‐enhanced ultrasound for real time monitoring of laparoscopic radiofrequency ablation for hepatocellular carcinoma: an observational pilot study. J Gastrointestin Liver Dis. 2019;28(4):457‐462. 31826072 10.15403/jgld-263

[15] Eun HS , Lee BS , Kwon IS , et al. Advantages of laparoscopic radiofrequency ablation over percutaneous radiofrequency ablation in hepatocellular carcinoma. Dig Dis Sci. 2017;62(9):2586‐2600. 28744835 10.1007/s10620-017-4688-6

[16] Badea R , Ciobanu L . Contrast enhanced and Doppler ultrasound in the characterization of the microcirculation. Expectancies and performances. Med Ultrason. 2012;14(4):307‐317. 23243644

[17] Dietrich CF , Nolsøe CP , Barr RG , et al. Guidelines and good clinical practice recommendations for contrast‐enhanced ultrasound (CEUS) in the liver‐update 2020 WFUMB in cooperation with EFSUMB, AFSUMB, AIUM, and FLAUS. Ultrasound Med Biol. 2020;46(10):2579‐2604. 32713788 10.1016/j.ultrasmedbio.2020.04.030

[18] Takayama T , Hasegawa K , Izumi N , et al. Surgery versus radiofrequency ablation for small hepatocellular carcinoma: a randomized controlled trial (SURF trial). Liver Cancer. 2021;11(3):209‐218. 35949295 10.1159/000521665 PMC9218617

[19] Berber E . The first clinical application of planning software for laparoscopic microwave thermosphere ablation of malignant liver tumours. HPB (Oxford). 2015;17(7):632‐636. 25980481 10.1111/hpb.12423 PMC4474511

[20] Abreu de Carvalho LF , Logghe B , Van Cleven S , et al. Local control of hepatocellular carcinoma and colorectal liver metastases after surgical microwave ablation without concomitant hepatectomy. Langenbecks Arch Surg. 2021;406(8):2749‐2757. 34076718 10.1007/s00423-021-02219-4

[21] Nieuwenhuizen S , Puijk RS , van den Bemd B , et al. Resectability and ablatability criteria for the treatment of liver only colorectal metastases: multidisciplinary consensus document from the COLLISION trial group. Cancers (Basel). 2020;12(7):1779. 32635230 10.3390/cancers12071779 PMC7407587

[22] Ito K , Takemura N , Inagaki F , Mihara F , Kokudo N . Difference in treatment algorithms for hepatocellular carcinoma between world's principal guidelines. Glob Health Med. 2020;2(5):282‐291. 33330822 10.35772/ghm.2020.01066 PMC7731415

[23] Liang P , Yu J , Lu MD , et al. Practice guidelines for ultrasound‐guided percutaneous microwave ablation for hepatic malignancy. World J Gastroenterol. 2013;19(33):5430‐5438. 24023485 10.3748/wjg.v19.i33.5430 PMC3761095

[24] Yu J , Cheng ZG , Han ZY , et al. Period‐dependent survival benefit of percutaneous microwave ablation for hepatocellular carcinoma: a 12‐year real‐world, Multicentric Experience. Liver Cancer. 2022;11(4):341‐353. 35978603 10.1159/000522134 PMC9294937

[25] Crocetti L , de Baére T , Pereira PL , Tarantino FP . CIRSE standards of practice on thermal ablation of liver Tumours. Cardiovasc Intervent Radiol. 2020;43(7):951‐962. 32382856 10.1007/s00270-020-02471-z

[26] Cao S , Zou Y , Lyu T , et al. Long‐term outcomes of combined transarterial chemoembolization and radiofrequency ablation versus RFA monotherapy for single hepatocellular carcinoma ≤3 cm: emphasis on local tumor progression. Int J Hyperthermia. 2022;39(1):1‐7. 34937501 10.1080/02656736.2021.1998660

[27] Vogl TJ , Nour‐Eldin NA , Hammerstingl RM , Panahi B , Naguib NNN . Microwave ablation (MWA): basics, technique and results in primary and metastatic liver neoplasms – review article. Rofo. 2017;189(11):1055‐1066. 28834968 10.1055/s-0043-117410

